# The development of meibomian glands in mice

**Published:** 2010-06-18

**Authors:** Chyong Jy Nien, Salina Massei, Gloria Lin, Hongshan Liu, Jerry R. Paugh, Chia-Yang Liu, Winston Whei-Yang Kao, Donald J. Brown, James V. Jester

**Affiliations:** 1 Gavin Herbert Eye Institute, University of California Irvine, CA; 2 Ophthalmology Department, University of Cincinnati, OH; 3 Southern California College of Optometry, Fullerton, CA

## Abstract

**Purpose:**

The purpose of this study was to characterize the natural history of meibomian gland morphogenesis in the mouse.

**Methods:**

Embryonic (E) and post natal (P) C57Bl/6 mouse pups were obtained at E18.5, P0, P1, P3, P5, P8, P15, and P60. Eyelids were fixed and processed for en bloc staining with Phalloidin/DAPI to identify gland morphogenesis, or frozen for immunohistochemistry staining with Oil red O (ORO) to identify lipid and antibodies specific against peroxisome proliferator-activated receptor gamma (PPARγ) to identify meibocyte differentiation. Samples were then evaluated using a Zeiss 510 Meta laser scanning confocal microscope or Nikon epi-fluorescent microscope. Tissues from adult mice (2 month-old) were also collected for western blotting.

**Results:**

Meibomian gland morphogenesis was first detected at E18.5 with the formation of an epithelial placode within the fused eyelid margin. Invagination of the epithelium into the eyelid was detected at P0. From P1 to P3 there was continued extension of the epithelium into the eyelid. ORO and PPARγ staining was first detected at P3, localized to the central core of the epithelial cord thus forming the presumptive ductal lumen. Ductal branching was first detected at P5 associated with acinar differentiation identified by ORO and PPARγ staining. Adult meibomian glands were observed by P15. Western blotting of meibomian gland proteins identified a 50 kDa and a 72 kDa band that stained with antibodies specific to PPARγ.

**Conclusions:**

We have demonstrated that meibomian gland development bears distinct similarities to hair development with the formation of an epithelial placode and expression of PPARγ co-incident with lipid synthesis and meibocyte differentiation.

## Introduction

Meibomian glands are greatly enlarged modified sebaceous glands without a hair follicle [1] and as a skin appendage, their development is thought to share similarity with hair and sebaceous gland morphogenesis in the skin. During development of pilosebeaceous units, mesenchymal-epithelial interactions at embryonic day 14.5 (E14.5) lead to the formation of an ectodermal-epithelial placode that progressively undergoes proliferation and growth to form hair follicles that break the skin surface after birth [2]. Sebaceous gland precursors appear to form along the upper segment of the hair follicle toward the end of embryogenesis and are characterized by the expression of peroxisome proliferator-activated receptor gamma (PPARγ) and the synthesis of lipid [3]. PPARs play critical roles in cell proliferation, differentiation, and apoptosis [4]. They are implicated in fetal maturation of murine epidermis, regulating keratinocyte differentiation, and the morphogenesis of skin appendages including hair follicles and sebaceous glands. The PPARγ isoform is the most studied receptor and is thought to be a key regulator of adipogenesis and adipocyte differentiation [5,6]. PPARγ is also required for the differentiation of the sebaceous gland [7] and has been shown to be present in mouse and human skin sebaceous glands in vivo [8].

By comparison, little is known about meibomian gland development [9,10] and the most extensive study, reported by Andersen, et al. [11], focuses on the human meibomian gland. More importantly, no studies have reported on the expression of PPARγ during meibomian gland development and its association with lipidogenesis.

Recently, a role for PPARγ in meibomian gland function was hypothesized based on findings that PPARγ localization undergoes specific age-related changes in older mouse meibomian glands [12]. In young mice (<6 months of age), PPARγ is localized in small vesicles within the cytoplasm and the nucleus that is associated with a high meibocyte proliferation index. Aging mice (>1 year of age) show primarily a nuclear localization that is associated with decreased meibocyte proliferation and meibomian gland atrophy. Overall, these findings suggest that altered PPARγ receptor signaling in older mice may control changes in cell cycle entry/proliferation, lipid synthesis and gland atrophy during aging that may underlie age-related meibomian gland dysfunction.

In this report we provide evidence that meibomian gland development bears distinct similarities to sebaceous gland development and that PPARγ expression coincides with lipogenesis and meibocyte differentiation. Overall these findings support the hypothesis that PPARγ is a major regulator of meibomian gland function.

## Methods

### Animals

C57Bl/6 mice were used in this study. Embryonic (E) and postnatal (P) mouse pups were obtained at E18.5, P0, P1, P3, P5, P8, P15, and P60. Four mice were sacrificed at each time point.

### Confocal microscopy

Eyelids from E18.5, P0, P3, P5, P8, and P15 were dissected and fixed in 4% paraformaldehyde overnight at 4 °C. Samples were then washed 2 times for 30 min each with 50% TD buffer (0.5% DMSO, 0.5% Triton X, 2.5% Dextran 40 in PBS, PH 7.4) at 4 °C. Tissue was then stained en bloc with Rhodamine Phalloidin (dilution 1:20 in 50% TD buffer; Molecular Probes, Invitrogen, Carlsbad, CA) and DAPI (4',6-diamidino-2-phenylindole nuclei stain, dilution 1:1,000; Molecular Probes, Invitrogen, Carlsbad, CA) overnight on a rocker at 4 °C. The next day, samples were washed with 50% TD buffer, 3 times, 30 min each, transferred to PBS, and scanned using a confocal microscope (LSM 510; Carl Zeiss MicroImaging, Thornwood, NY) using 543 nm and 633 nm lasers under a 40× oil objective.

### Inmunohistochemistry

Eyelids were fixed in 2% PFA overnight, placed in 15% sucrose for 4 h and then transferred to 30% sucrose overnight. Lids were embedded in Tissue-Tek® embedding medium for frozen tissue (Sakura Finetek USA, Inc, Torrance, CA), frozen in liquid nitrogen and blocks stored at −80 °C until sectioned on a cryostat. Cryosections (8 μm thick) were then cut mounted on glass slides, and either stained for Oil Red O (ORO, Sigma-Aldrich, St. Louis, MO) or with antibodies to PPARγ. Sections were stained with ORO by first rinsing in PBS and then incubating in 0.5% ORO solution for 10 min. Sections were then rinsed with PBS and counterstained with hematoxylin.

For immunostaining, tissue sections were rehydrated with PBS, placed in acetone for 3 min and then re-hydrated in PBS. Sections were then blocked with 1% BSA in PBS for 30 min at 37 °C and then stained with rabbit anti-PPARγ (1:50; Abcam, Cambridge, MA) for 1 h at 37 °C. Sections were then washed with PBS, stained with FITC conjugated goat anti-rabbit IgG (dilution 1:200; Invitrogen, Carlsbad, CA) for 1 h at 37 °C and then counterstained with DAPI. For negative controls, primary antibodies were omitted. The samples were then evaluated and imaged using a Nikon Eclipse E600 fluorescence microscope.

### Western blotting

Eyelids from P60 mice were removed and meibomian glands dissected from both the upper and lower eye lids. Tissue was weighed and 39 mg of meibomian glands from 5 mice were then placed in 0.5 ml of Radioimmunoprecipitation assay buffer (RIPA buffer, 25 mM Tris-HCl pH 7.6, 150 mM NaCl, 1% NP-40, 1% sodium deoxycholate, 0.1% SDS) containing protease inhibitor and phosphatase inhibitor. Tissue was centrifuged at 5,000× g for 20 min and the supernatant was collected. Total protein was then measured using the RC DC Protein Assay (Bio-Rad Laboratories, Hercules, CA). For comparison, samples from white and brown fat were also obtained and protein collected. White and brown fat tissue was frozen in liquid nitrogen and placed in 1 ml of RIPA buffer containing protease inhibitor and phosphatase inhibitor. The tissue samples were sonicated, then centrifuged at low speed 1,500× g for 5 min at 4 °C to obtain the supernatant. The supernatant was centrifuged again at 4 °C under the “Fast Cool” function of the centrifuge. Samples were run on 10% SDS PAGE and the proteins were transferred to PVDF membrane via iBlot Gel Transfer Device (Invitrogen). The blot was then blocked using 5% non-fat dry milk in TBS-T (0.2% Tween-20). Following blocking, the blot was immunostained using antibodies to PPARγ (1:500 Dilution) and rinsed with TBS-T (0.2% Tween-20). The blot was then incubated with goat anti-rabbit IgG (H^+^L) HRP (Dilution 1:2,500; Thermo Scientific, Rockford, IL) and rinsed with TBS-T (0.2% Tween-20). To visualize the bands, the blot was incubated with SuperSignal West Pico Chemiluminescent Substrate (Thermo Scientific, Rockford, IL) and detected using photographic film.

## Results

### Meibomian gland morphogenesis

**Figure 1:**
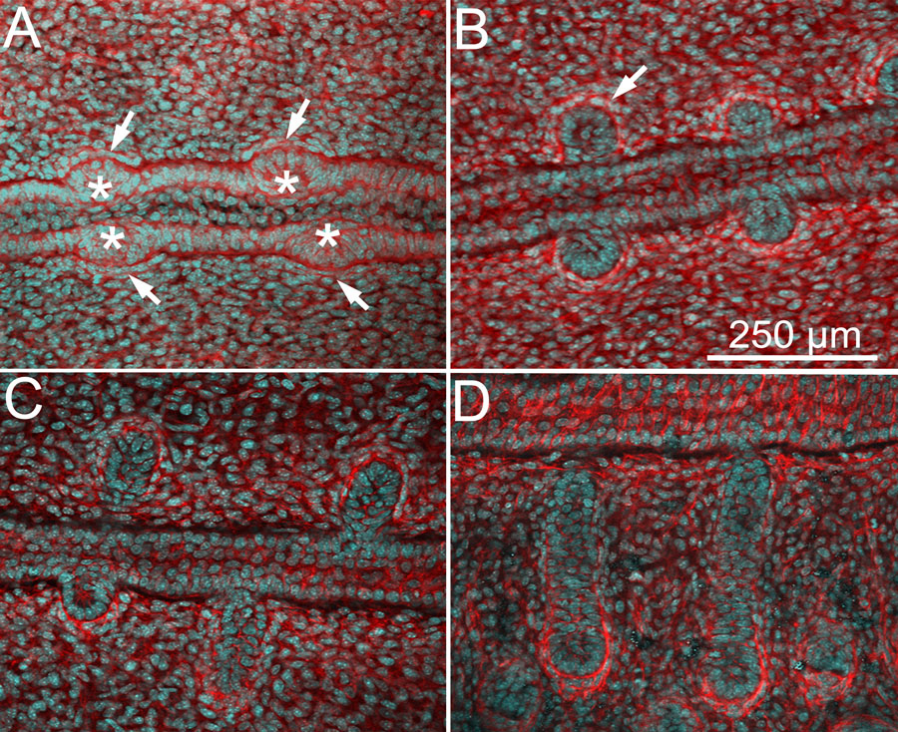
Confocal images using actin (red) and nuclei (DAPI, cyan) staining of eyelids at different time points of meibomian gland morphogenesis. At E18.5 (**A**), we observed the formation of an epithelial condensation (asterisk) within the fused lid margin. Epithelial placodes in the superior and inferior lids also appeared opposite to each other, and were associated with condensation of mesenchyme as detected by cell alignment and increased actin staining of mesenchyme directly adjacent to the placode (arrows). At P0 (**B**), we observed an epidermal invagination (arrow) that undergoes proliferation and maturation with progressive enlargement and elongation adopting a tubular shape at P1 and P3 (**C** and **D**, respectively).

Meibomian gland development appeared to start at E18.5 (Figure 1A) with the formation of an epithelial placode (Figure 1A; asterisk) within the fused eyelid margin epithelium. Epithelial placodes in the superior and inferior lids also appeared to form in tandem, immediately opposite to each other, and were associated with condensation of mesenchyme as detected by cell alignment and increased actin staining of mesenchyme directly adjacent to the placode (Figure 1A; arrows). At birth (P0, Figure 1B), the epithelial invagination into the eyelid mesenchyme was first detected and appeared to form a solid cord with no clear lumen (Figure 1B, arrow) and with more prominent condensation of mesenchyme surrounding the epithelial invagination. Progressive lengthening of the invaginating epithelial cord was detected from P1 (Figure 1C) to P3 (Figure 1D) with little additional morphogenetic changes.

**Figure 2:**
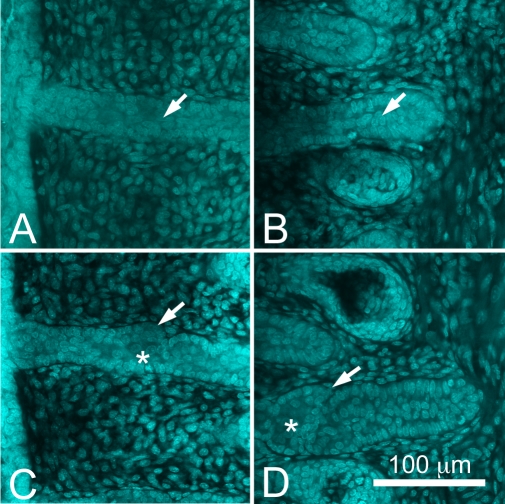
Nuclei staining (DAPI) at P3 and P5. At P3 (**A** and **B**), meibomian glands appeared to be comprised of a cord of epithelium with a bordering basal cell layer and a two-three cell suprabasal cell layer within the inner core at the distal end of the developing meibomian gland (**A**, arrow). At the proximal end there appeared to be a reduction of the number of cells within the inner core of the developing duct (**B**, arrow). By P5 (**C** and **D**), early branching of the invaginating epithelial cord was observed (arrows). Additionally, there appeared to be enlargement of cells within the central cord of the epithelium (asterisk).

**Figure 3:**
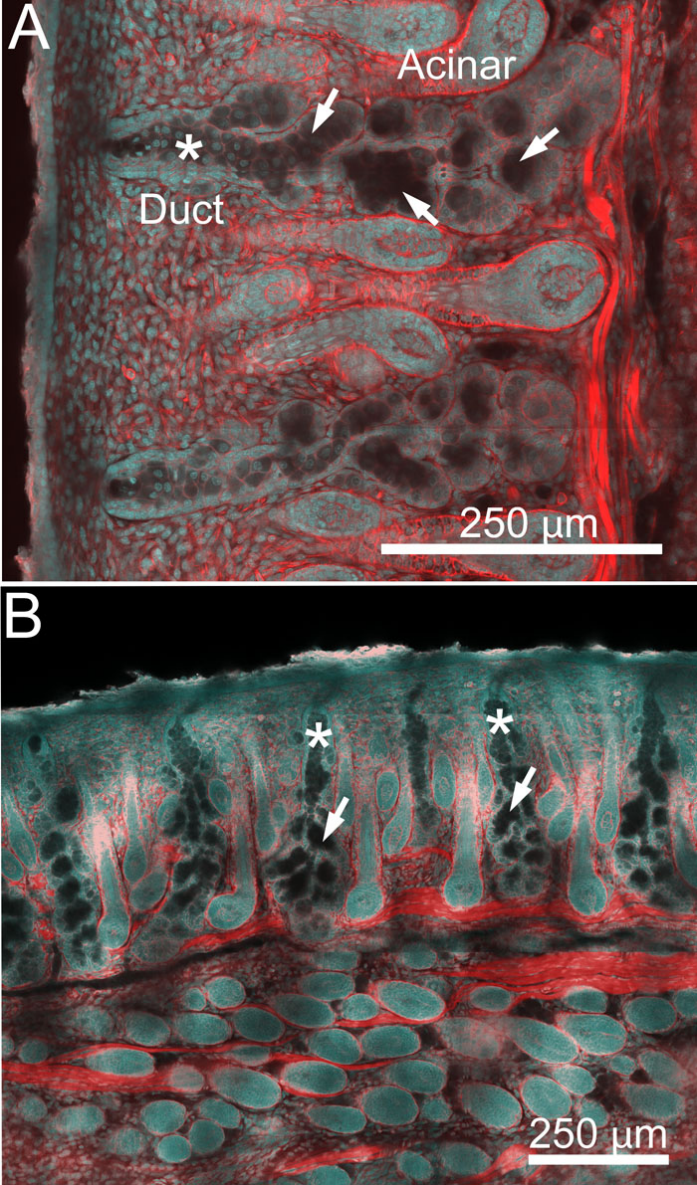
Confocal images using actin (red) and nuclei (DAPI, cyan) staining of eyelids at P8 and P15. At P8 (**A**), developing meibomian glands showed distinct differentiation into ductal and acinar regions. Within the ductal region, basal epithelial cells along with a single layer of suprabasal cells appear to line a more central region containing enlarged epithelial cells that lacked actin staining (asterisk). Within the acinar region, the developing meibomian gland duct branched into multiple ductules that appeared to lack any cellular contents (arrows) and terminated into developing acini. At P15 (**B**), the meibomian glands appear to obtain an adult meibomian gland morphology.

At higher magnification, developing meibomian glands at P3 appeared to be comprised of a cord of epithelium with a bordering basal cell layer and a two-three cell suprabasal cell layer within the inner core at the distal end of the developing meibomian gland (Figure 2A, arrow). At the proximal end of the developing gland there appeared to be a reduction of the number of cells within the inner core of the developing duct (Figure 2B, arrow). By P5, early branching of the invaginating epithelial cord was detected at both the distal (Figure 2C, arrow) and proximal end (Figure 2D, arrow) of the developing gland. Additionally, there appeared to be enlargement of cells within the central cord of the epithelium (Figure 2C,D, asterisk). By P8 the developing meibomian glands showed distinct differentiation into ductal and acinar regions (Figure 3A). Within the ductal region, basal epithelial cells along with a single layer of suprabasal cells appeared to line a more central region containing enlarged epithelial cells that lacked actin staining (Figure 3A, asterisk). Within the acinar region, the developing meibomian gland duct branched into multiple ductules that appeared to lack any cellular contents (arrows) and terminated into developing acini. By P15, the meibomian glands appeared to obtain a normal meibomian gland morphology (Figure 3B).

### Meibocyte differentiation and lipid synthesis

**Figure 4:**
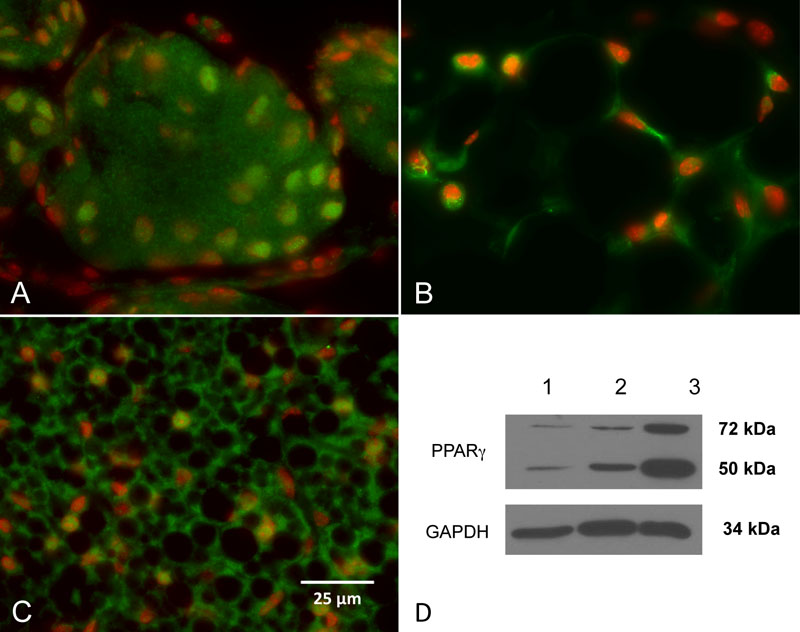
PPARγ (Green) and nuclei (DAPI, red) staining of meibomian glands, white fat, and brown fat from adult, P60 mice. In the meibomian glands (**A**), nuclear and extra-nuclear staining of the plasma membrane and intracellular structures, possibly microsomes were detected. Staining of white fat and brown fat (**B** and **C**, respectively) showed predominantly nuclear staining. Western blotting of meibomian gland protein with PPARγ antibodies identified a 50 kDa protein band (**D**, lane 1) that was also present in extracts from white fat and brown fat (**D**, lane 2 and 3, respectively). Note that the meibomian gland, white fat and brown fat all showed a second band with a molecular weight of 72 kDa that may represent post-translational modifications of PPARγ.

Meibocyte differentiation was evaluated by staining for PPARγ using a rabbit polyclonal antibody directed against a synthetic peptide derived from residues 50–150 of human PPARγ. Immunostaining of meibomian glands from adult, P60 mice showed nuclear and extra-nuclear staining of the plasma membrane and intracellular structures, possibly microsomes (Figure 4A). Staining of white fat and brown fat (Figure 4B,C, respectively) showed predominantly nuclear staining. Western blotting of meibomian gland protein from adult mice with PPARγ antibodies identified a 50 kDa protein band (Figure 4D, lane 1) that was also present in extracts from white fat and brown fat (Figure 4D, lane 2 and 3, respectively). An additional band at 72 kDa was also identified that might be related to post-translational modifications of the protein.

**Figure 5:**
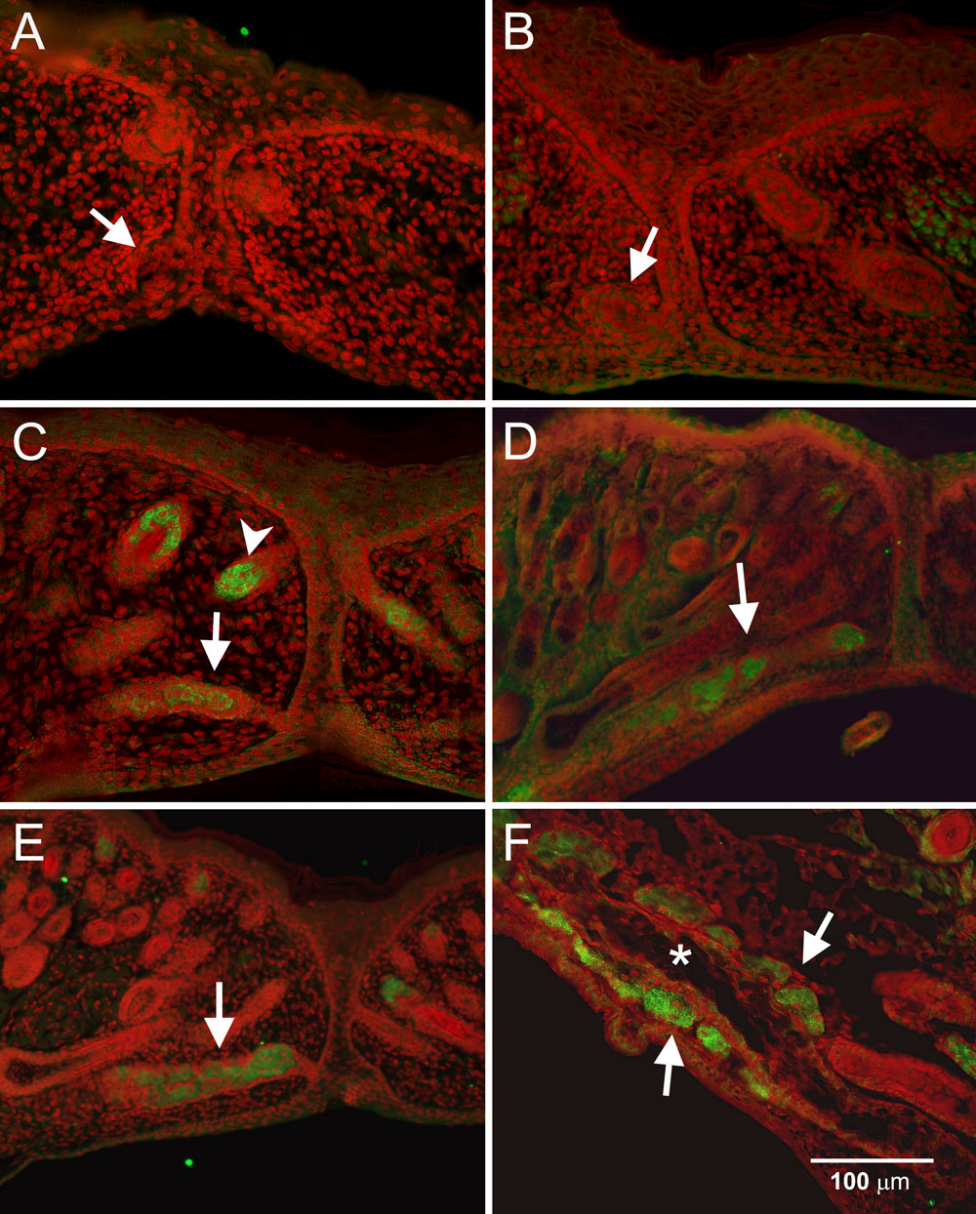
PPARγ (green) and nuclei (DAPI, red) staining during meibomian gland morphogenesis. At E18.5 (**A**) and P0 (**B**), PPARγ staining of the epithelial placode was negative. At P3 (**C**), PPARγ was first detected in the lumen of the developing gland and increased progressively as the gland continued to develop at P5 (**D**) and P8 (**E**). At P15 (**F**), PPARγ staining was limited to the acini (arrows) or the terminal regions of the branching ductules (the asterisk shows a well developed central duct at P15).

During morphogenesis, staining for PPARγ was negative at E18.5 and at birth (P0) (Figure 5A,B, respectively). PPARγ staining was first detected at P3 within the central region of the invaginating epithelium (Figure 5C, arrow); the same time that sebaceous glands appeared to stain for PPARγ (arrowhead). PPARγ staining appeared to progressively increase at P5 and P8 as the gland continued to develop (Figure 5D,E, arrow), however the localization appeared to be both within and along the proximal end of developing meibomian gland duct. By P15, PPARγ staining was localized to the meibomian gland acini (Figure 5F, arrows) that bordered the central ductal epithelium, which was negative for PPARγ (Figure 5F, asterisk).

**Figure 6:**
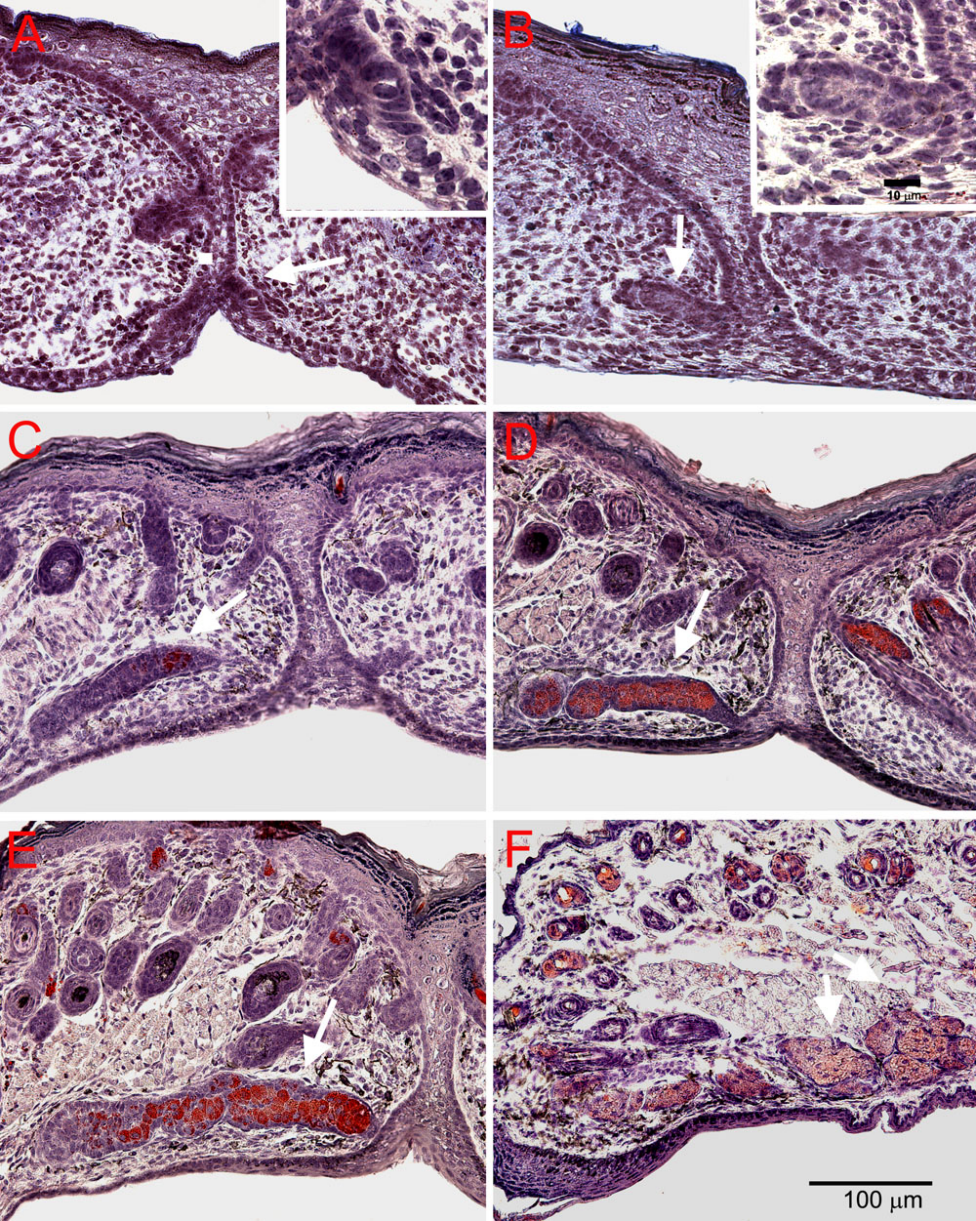
ORO staining for lipid during meibomian gland development at different time points. Consistent with PPARγ, ORO staining was absent at E18.5 (**A**) and P0 (**B**). Lipid production was first detected at P3 (**C**) within the central region of the developing duct. At P5 (**D**) and P8 (**E**), ORO staining continued to be limited to the inner core of the developing duct. After eyelid opening at P15 (**F**), ORO was present in the acini of the mature gland.

Association of meibocyte differentiation with lipid production was assessed by ORO staining and showed that eyelids were negative for lipid at both E18.5 and P0 (Figure 6A,B, arrow). Lipid was first detected at P3 and was limited to the central region of the invaginating epithelium (Figure 6C, arrow); the same region stained by antibodies to PPARγ. At P5 and P8, ORO staining continued to be limited to the inner core of the developing duct and ductules (Figure 6D,E, respectively). After eyelid opening at P15, ORO was present in the acini of the mature meibomian glands (Figure 6F).

## Discussion

To our knowledge, reports covering the development of the meibomian gland are fragmentary and limited, for the most part, to the appearance of tarsal glands during eyelid opening [9,10]. The most extensive study reported by Andersen et al. [11] and also discussed by Knop et al. [13] reports that meibomian glands in the human eyelid initially form as epithelial cords that develop following eyelid fusion. Lateral branching of the epithelial cords then form acini later in development. Lipid synthesis was also noted to occur within the acini and the epithelial invagination thus forming the central ductal system of the glands.

Our results with confocal microscopy support and extend these observations and indicate that meibomian gland development in the mouse is initiated around E18.5 with the formation of an epithelial placode and mesenchymal condensation, similar to that observed in hair follicle development [14,15]. Invagination of the epithelium into the developing mesenchyme then continues from birth to about P3 with initial branching of the epithelial cord detected at P5. By P8 the developing meibomian gland shows extensive ductal branching and the formation of distinct acini with mature meibomian glands present by P15 or eyelid opening.

During meibomian gland development, PPARγ expression was first detected at P3, before epithelial branching and the formation of distinct acini. PPARγ expression was also localized to the central core of the invaginating epithelium, the same region where lipid synthesis was first detected. Interestingly, PPARγ expression remained localized to the developing duct and acinar regions from P5 to P8, but was restricted to the acini by P15. Overall, these findings suggest that PPARγ plays an important role in meibomian gland morphogenesis, meibocyte differentiation and lipid synthesis.

Interestingly, western blots of adult meibomian gland tissue extracts identified a low and high molecular weight PPARγ protein. Message for PPARγ can be alternatively spliced to a 475 amino acid (γ1) and 505 amino acid (γ2) isoform of approximately 50 kDa. PPARγ also contains a consensus serine phosphorylation site at S112 that may be associated with decreased transcriptional activity [16,17] and two sumoylation sites, K107 and K395, that may be linked to nuclear-cytoplasmic transport, apoptosis, and transcriptional regulation [18]. Since sumoylation covalently attaches sumo-1 or sumo-2/3, each approximately 11 kDa, it is likely that the high molecular weight PPARγ identified in this study represents a post translational sumoylation. Clearly, future studies identifying the post translational modifications of PPARγ will be important to understanding the role of this receptor in regulating meibomian gland function.

Additionally, PPARγ contains an NH_2_-terminal transactivation domain (residues 30–136), a DNA binding domain (residues 136–140), and a COOH-terminal ligand-binding domain (residues 204–505) containing a ligand-dependent transactivation function [19]. Interestingly, PPARγ functions as a “molecular switch” and can recruit corepressors, N-CoR and SMRT, when unliganded PPARγ binds to some target genes promoters, which upon ligand binding recruits co-activators, SRC1/CBP and TRAP/DRIP/ARC complexes [20].

Importantly, PPARγ has been shown to play a critical role in both adipogenesis and sebaceous gland development with mice chimeric for PPARγ null cells showing little or no contribution to the formation of fat or sebaceous glands [7]. Additionally, PPARγ agonists, such as thiazolidinediones and rosiglitazone, increase sebum production in diabetic patients and lipid synthesis in sebocyte cell cultures, respectively [21]. Recent studies also suggest that there can be distinct gender differences in the transcriptional activity of the *PPAR* gene [22] and there is at least one report suggesting that androgens and PPAR ligands may act synergistically to regulate lipid synthesis in cultured sebocytes [23]. If transferable to the meibomian gland, these findings suggest that understanding the role of PPARγ in meibomian gland function may provide important insights into the pathogenesis of meibomian gland dysfunction. Further work studying the modulation of PPARγ expression or activity will be needed to clarify the role PPARγ in regulating meibum production. More interestingly, selective modulation of PPARγ activity may be a potential therapeutic strategy for the treatment of dry eye syndrome and meibomian gland dysfunction.

In summary, we have demonstrated that meibomian gland development starts with the formation of an epithelial placode similar to that observed for the development of the pilosebaceous unit. Development then proceeds with invagination of the epithelium into the eyelid mesenchyme forming a solid cord. By post natal day 3, the adipogenic nuclear receptor PPARγ is first expressed within the epithelial cord in association with lipid synthesis, thus forming the presumptive ductal lumen, and later, the nascent acini at post natal day 5. Meibomian glands appear to be completely formed by the time of eyelid opening with PPARγ restricted to the acinar compartment. Overall, PPARγ appears to be a molecular marker of meibocyte differentiation.
